# Essential oils as bioherbicides: effects of orange and citronella oils on weed germination and early growth

**DOI:** 10.1002/ps.71009

**Published:** 2026-06-12

**Authors:** Geovana Rocha Marzochi, Leonardo de Oliveira Semensato, Francielli Santos de Oliveira, Virginia Claudia Paulino Silva, Eliane Gomes Fabri, Evandro Henrique Schinor, Andréia Cristina Silva Hirata, Moacir Rossi Forim, Angélica Correa Kauffmann, Patricia Andrea Monquero

**Affiliations:** ^1^ Center of Agricultural Sciences Federal University of São Carlos (UFSCar) Araras Brazil; ^2^ Department of Plants Soils and Climate – Utha State University Logan Utah USA; ^3^ Research and Development Center Agronomic Institute of Campinas Campinas Brazil; ^4^ São Paulo Regional Agency for Agribusiness Technology Presidente Prudente Brazil; ^5^ Department of Chemistry Federal University of São Carlos (UFSCar) São Carlos Brazil

**Keywords:** allelopathy, weed, essential oil, bioherbicide

## Abstract

**BACKGROUND:**

Increasing selection of herbicide‐resistant weed biotypes has intensified the search for alternatives to the intensive use of synthetic herbicides. Among these alternatives, essential oils (EO) have attracted attention because of their allelopathic potential and low environmental impact. This study evaluated the effects of different doses of EOs extracted from orange (*Citrus sinensis*) by hydrodistillation and steam distillation, and from citronella (*Cymbopogon nardus* and *C*. *winterianus*), on the germination and early development of *Eleusine indica*, *Digitaria insularis*, *Euphorbia heterophylla*, and *Amaranthus hybridus*.

**RESULTS:**

The EOs analyzed were characterized primarily by oxygenated monoterpenes and terpene hydrocarbons, notably citronellol, citronellal, geraniol and limonene. Citronella EOs caused strong inhibition of germination and vigor, resulting in near‐total seedling suppression from 10 mL L^−1^ onward. By contrast, orange EOs exhibited moderate effects, with partial and dose‐dependent reductions in germination and early growth, and the weed species differed in their susceptibility to the EOs.

**CONCLUSIONS:**

The combined results indicate that citronella EOs exhibit high bioactivity and stand out as promising candidates for bioherbicide development, whereas orange EOs show a less intense but relevant allelopathic effect, supporting their potential use as complementary tools in sustainable weed management strategies. © 2026 The Author(s). *Pest Management Science* published by John Wiley & Sons Ltd on behalf of Society of Chemical Industry.

## Graphical abstract

Essential oils from orange and citronella showed dose‐dependent inhibition of weed germination and early growth, highlighting their potential as bioherbicides and sustainable tools for integrated weed management.

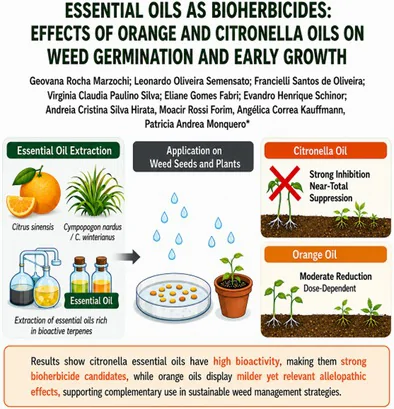

## 1 INTRODUCTION

Chemical control based on synthetic herbicides is the most widely adopted method for weed management because of its efficiency and practicality.^1^ However, the intensive and repeated use of these products has generated significant agronomic and environmental impact. Between 2010 and 2020, herbicide consumption in Brazil increased from 157 500 to 329 700 tons of active ingredients per year, while the cultivated agricultural area expanded by only 24%. This discrepancy highlights the growing dependence on chemical inputs and the declining effectiveness of glyphosate.^2^

This scenario has favored the selection of resistant biotypes and tolerant weed species, compromising the sustainability of production systems. In Brazil, glyphosate resistance represents one of the main phytosanitary challenges, with more than 20 confirmed cases involving at least 12 species.^3^ In addition, resistance has been reported to herbicides with different modes of action, such as acetolactate synthase (ALS), acetyl‐CoA carboxylase (ACCase), photosystem II and protoporphyrinogen oxidase (PROTOX) inhibitors, as well as multiple resistance in species such as *Euphorbia heterophylla*, *Amaranthus hybridus*, *Eleusine indica* and *Digitaria insularis*.^3^, ^4^

Given this context, the development of alternative weed management strategies that reduce environmental impact while maintaining control efficacy is essential. Among these alternatives, essential oils (EO) have gained attention as natural compounds with allelopathic activity, low environmental impact and reduced toxicity, showing promising results as bioherbicides.^5^, ^6^, ^7^, ^8^

Previous studies have demonstrated that EOs can inhibit weed germination and early growth, although their effectiveness depends on species, phenological stage and applied concentration.^6^, ^9^, ^10^, ^11^, ^12^, ^13^, ^14^ Citronella and citrus EOs, particularly orange oil, have been highlighted for their ability to significantly reduce weed germination and early growth, reinforcing their potential as complementary tools in sustainable weed management.^7^, ^15^ There are different methods for extracting EOs, and differences in extraction methods, such as hydrodistillation (LH) and steam distillation (LV), may result in distinct EO compositions and, consequently, influence their biological activity. However, challenges such as variability in chemical composition, high volatility and limitations in formulation and large‐scale application still restrict the broader use of EOs in agriculture.^16^

Thus, this study aimed to evaluate the pre‐ and post‐emergence effects of sweet orange and citronella EOs on *Eleusine indica*, *D. insularis*, *Euphorbia heterophylla* and *A. hybridus*, aiming to identify efficient weed control alternatives with reduced environmental impact.

## 2 MATERIALS AND METHODS

The study was conducted at the Center for Agricultural Sciences of the Federal University of São Carlos (CCA/UFSCar), Araras campus, São Paulo, Brazil (22° 21′ 25″ S, 47° 23′ 02″ W). The experiments were performed under controlled greenhouse conditions and in the Laboratory of Ecotoxicology and Environmental Chemistry at CCA/UFSCar, as well as in the Chemistry Laboratory at UFSCar.

### 2.1 Essential oils and extraction methods

EOs from orange (*Citrus sinensis*) and two species of citronella [*Cymbopogon nardus* (Ceylon citronella) and *C*. *winterianus* (Java citronella)] were used. Sweet orange fruits were collected between August and September at the ‘Sylvio Moreira’ Citrus Research Center (IAC), Cordeirópolis, Brazil. One orange EO was obtained by hydrodistillation using a Clevenger‐type apparatus with 300 g of fresh peel and 1 L of distilled water, distilled for 2–3 h after the first condensate drop. A second orange EO was obtained by steam distillation and supplied by Cisol do Brasil Exportação LTDA (Artur Nogueira, Brazil).

Citronella EOs (*C. nardus* and *C*. *winterianus*) were extracted from fully expanded young leaves collected between September and October at the IAC experimental field in Campinas, São Paulo. Approximately 200 g of leaves were subjected to hydrodistillation in a Clevenger‐type apparatus for approximately 1 h. All EO were stored in amber vials at −18 °C until bioassays were conducted.

### 2.2 Chromatographic analysis

Chromatographic analyses were performed for each EO used in the study, aiming to identify and quantify the constituent compounds, particularly the major components potentially. The EOs were diluted in hexane 0.1% (v/v) prior to chromatographic analysis. Volatile compounds were characterized by gas chromatography, with identification based on mass spectra comparison using the NIST 17.0 library and literature data reported.^17^ Retention indices were calculated according to the method of Van den Dool and Kratz.^18^

### 2.3 Osmotic potential determination

The osmotic potential of the EO was determined using an osmometer (μosm kg^−1^) and converted to MPa.^19^ Polyethylene glycol 6000 (PEG‐6000) solutions were prepared to simulate equivalent osmotic potentials^19^ and used in germination assays to discriminate osmotic effects from biochemical effects of the EOs.

### 2.4 Experimental design and treatments

Experiments were conducted in a completely randomized design with four replications, in 4 × 5 factorial arrangements, evaluating four EOs (orange steam distillation, orange hydrodistillation, Ceylon citronella hydrodistillation and Java citronella hydrodistillation) and five concentrations (0, 10, 25, 40, and 60 mL L^−1^), with 0 mL L^−1^ as the control. Tween 80 (0.5%) was added as a surfactant.

The weed species evaluated were *Eleusine indica*, *D. insularis*, *Euphorbia heterophylla* and *A. hybridus*. Seeds were purchased from Agrocosmos (Engenheiro Coelho, Brazil), surface‐sterilized in 0.1% NaClO for 2 min, and air‐dried on filter paper.

### 2.5 Germination bioassay

For germination evaluation, 50 seeds per species were placed in 9‐cm Petri dishes containing Germitest paper moistened with 2.5 times its weight in EO solutions (0, 10, 25, 40 and 60 mL L^−1^). Plates were incubated in a biochemical oxygen demand (BOD) chamber at 25 °C with a 12‐h photoperiod.^20^ Germination was recorded daily for 15 days, considering seeds germinated when radicle protrusion ≥2 mm occurred. Total germination percentage and germination speed index (GSI) were calculated according to Wardle *et al*.^21^

### 2.6 Early growth bioassay

Seeds were germinated in sterilized containers with Germitest paper in a BOD chamber (25 °C; 12 h photoperiod). After radicle protrusion ≥2 mm, ten uniform seedlings per replication were selected following Voltarelli *et al*.^22^ and treated with 2.5 mL of each EO solution. After 7 days, root length (RL) and shoot length (SL) were measured using a ruler.

### 2.7 Post‐emergence bioassay

Seedlings were grown in pots containing dystrophic Red Latosol under greenhouse conditions. At the one‐ to two true‐leaf stage, EO were applied by spraying (~1.5 mL per pot) using a hand sprayer, ensuring leaf coverage without runoff. Visual phytotoxicity was assessed at 8 and 20 days after application (DAA) using a scale from 0% (no injury) to 100% (plant death).^23^ At 20 DAA, seedlings were measured for RL and SL, dried at 60 °C for 72 h, and weighed to determine dry biomass.^7^

### 2.8 Statistical analysis

Data were subjected to analysis of variance, with means compared by Tukey's test (*P* ≤ 0.05), using R software.^24^ In addition, regression analysis was performed, selecting models with *R* ^2^ > 0.90 using SigmaPlot 15.^25^ Data were square‐root transformed and expressed as percentages relative to the control.^26^

## 3 RESULTS

### 3.1 Chromatographic analysis of essential oils

The biological responses observed in germination, growth and post‐emergence assays were closely related to the chemical composition of the EOs (Tables 1 and 2). Citronella EOs (CC and CJ) were rich in oxygenated monoterpenes, mainly citronellal, geraniol, citronellol and citral isomers. By contrast, orange EOs (LH and LV) were dominated by limonene and contained lower proportions of oxygenated constituents; 31 compounds were detected in LV and 14 in LH, which may explain their more moderate and concentration‐dependent phytotoxic effects. Overall, the higher bioactivity of citronella oils is directly linked to their enrichment in oxygenated monoterpenes, whereas the limonene‐dominated profile of orange oils confers lower phytotoxic potency.

**Table 1:** Chromatographic analysis of orange (*Citrus sinensis*) essential oils obtained by steam distillation (LV) and hydrodistillation (LH)

Compound	RI_exp_ (LV)	RI_exp_ (LH)	RI_lit_	Area (%) LV	Area (%) LH
*α*‐Pinene	931.64787	931.64787	932	0.23	0.24
*β*‐Thujene	971.77155	971.77155	971	0.21	0.20
Sabinene	976.24591	0.00	976	0.05	0.00
*β*‐Pinene	989.08694	989.01419	991	1.77	2.11
Myrcene	1003.48837	1003.99709	1003	1.33	0.29
*α*‐Phellandrene	1007.55814	1007.66715	1007	0.19	0.22
*α*‐Terpinene	1016.71512	0.00	1017	0.04	0.00
*p*‐Cymene	1024.45494	0.00	1024	0.11	0.00
*d*‐Limonene	1029.76017	1029.72384	1029	83.22	95.32
*γ*‐Terpinene	1057.99419	1058.21221	1058	0.61	0.04
Terpinolene	1071.07558	0.00	1071	0.54	0.00
Linalool	1085.06541	1085.24709	1085	0.11	0.05
(*E*)‐*β*‐Ocimene	1099.20058	1099.27326	1099	6.77	0.98
(*Z*)‐*β*‐Ocimene	1105.13878	0.00	1105	0.10	0.00
Limonene oxide	1137.28432	0.00	1136	0.03	0.00
Terpinen‐4‐ol	1152.10053	1152.40060	1152	0.47	0.14
*α*‐Terpineol	1180.79520	0.00	1180	0.32	0.00
Neral	1195.31133	1195.61140	1195	1.01	0.10
Decanal	1206.10169	1206.36535	1206	0.50	0.25
Citronellol	1225.80038	0.00	1226	0.31	0.00
Geranial	1237.92844	0.00	1238	0.43	0.00
Carvone	1249.90584	0.00	1250	0.11	0.00
(*E*)‐Carveol	1267.60829	0.00	1267	0.47	0.00
(*Z*)‐Carveol	1271.86441	0.00	1271	0.09	0.00
Perillyl alcohol	1276.64783	0.00	1278	0.41	0.00
Perillaldehyde	1291.33710	0.00	1290	0.12	0.00
*α*‐Copaene	1376.94656	1376.90840	1377	0.07	0.04
*β*‐Caryophyllene	1421.62480	0.00	1420	0.00	0.00
*β*‐Elemene	0.00	1388.74046	1387	0.00	0.02
*α*‐Humulene	1431.27943	0.00	1431	0.06	0.00
Valencene	1494.11303	0.00	1494	0.14	0.00
*δ*‐Cadinene	1519.18367	0.00	1518	0.11	0.00
Total				100	100

*Note*: RI_exp_, experimental retention index calculated using a homologous series of *n*‐alkanes; RI_lit_, retention index reported in the literature (Adams, 2007)^17^. LV, essential oil obtained by steam distillation; LH, essential oil obtained by hydrodistillation. Percentages correspond to relative peak areas obtained by gas chromatography–mass spectrometry without correction factors.

**Table 2:** Chemical composition (% relative area) of Ceylon citronella (CC; *Cymbopogon nardus*) and Java citronella (CJ; *Cymbopogon winterianus*) essential oils determined by gas chromatography–mass spectrometry

Compound	RI_exp_ (CC)	RI_exp_ (CJ)	RI_lit_	Area (%) CC	Area (%) CJ
*α*‐Thujene	985.30375	0.00	985	0.15	0.00
*α*‐Pinene	989.19607	989.05056	989	0.15	0.15
Camphene	1004.14244	0.00	1005	0.09	0.00
Limonene	1028.67006	1028.67006	1030	7.03	6.83
*α*‐Terpinene	0.00	1053.37936	1053	0.00	0.10
Sabinene	1032.23110	0.00	1035	0.03	0.00
Myrcene	1034.15698	0.00	1035	0.05	0.00
*α*‐Phellandrene	1036.08285	0.00	1037	0.32	0.00
3‐Carene	1046.62064	0.00	1050	0.16	0.00
*α*‐Terpinene	1048.94622	1053.37936	1053	0.08	0.10
*p*‐Cymene	1053.19767	1058.06686	1060	0.46	0.03
*γ*‐Terpinene	1071.69331	0.00	1065	0.29	0.00
Linalool	1099.27326	1099.38227	1097	0.82	0.78
(*Z*)‐Rose oxide	1105.40135	0.00	1104	0.09	0.00
Hotrienol	1109.78995	1110.12753	1110	0.20	0.05
Limonene‐1,2‐epoxide	1126.21905	0.00	1125	0.08	0.00
(*Z*)‐Verbenol	1127.83196	1128.28207	1125	0.22	0.05
(*E*)‐Pinocarveol	1138.40960	0.00	1135	0.04	0.00
neo‐Isopulegol	1143.58590	0.00	1146	1.05	0.00
Isopulegol	1148.68717	1148.79970	1148	1.55	0.68
Citronellal	1152.47562	1153.45086	1153	26.19	33.57
(*E*)‐Isopulegol	1158.85221	1159.03976	1156	0.22	0.38
Isoborneol	0.00	1169.99250	1168	0.00	0.04
Menthone	1161.25281	0.00	1161	0.28	0.00
Terpinen‐4‐ol	1179.81995	1181.09527	1184	0.53	0.04
*α*‐Terpineol	1194.59865	1195.87397	1194	0.12	0.06
*γ*‐Terpineol	1206.10169	1206.47834	1205	0.62	0.11
(*Z*)‐Carveol	1220.79096	0.00	1218	0.04	0.00
Citronellol	1225.95104	1226.40301	1226	12.35	13.28
Neral (citral B)	1237.89077	1238.15443	1238	6.83	0.41
Geraniol	1249.94350	1250.35782	1253	22.11	16.28
Geranial (citral A)	1267.49529	1267.60829	1264	9.19	0.57
Citronellyl formate	1297.74011	0.00	1296	0.07	0.00
*δ*‐Elemene	1339.19847	1339.31298	1340	0.10	0.12
*α*‐Cubebene	1348.24427	1348.39695	1349	1.30	3.05
*γ*‐Elemene	0.00	1351.75573	1340	0.00	0.56
*α*‐Copaene	1370.57252	1376.90840	1376	0.15	3.24
*β*‐Bourbonene	1376.83206	1382.74809	1383	0.34	0.10
*β*‐Elemene	1385.07634	1385.15267	1387	0.17	0.20
*α*‐Ylangene	1390.07634	1390.19084	1390	0.20	2.31
*β*‐Caryophyllene	1421.27159	1421.31083	1422	1.90	0.16
*α*‐Bergamotene	0.00	1431.51491	1430	0.00	0.05
Aromadendrene	0.00	1446.78179	1442	0.00	0.06
allo‐Aromadendrene	0.00	1450.78493	1450	0.00	0.03
*α*‐Humulene	0.00	1457.45683	1452	0.00	0.11
*β*‐Farnesene	0.00	1463.77551	1459	0.00	0.04
*γ*‐Muurolene	0.00	1473.31240	1473	0.00	0.04
*ar*‐Curcumene	0.00	1476.09890	1476	0.00	0.19
*α*‐Humulene	1457.41758	0.00	1452	0.22	0.00
Germacrene D	1482.73155	1482.81005	1480	0.31	2.13
*β*‐Selinene	0.00	1491.13030	1489	0.00	0.04
*α*‐Selinene	0.00	1493.75981	1494	0.00	0.05
*δ*‐Muurolene	0.00	1497.17425	1496	0.00	0.13
Bicyclogermacrene	0.00	1499.33281	1499	0.00	0.46
Germacrene A	0.00	1509.30612	1508	0.00	0.19
*γ*‐Cadinene	1514.53061	1514.61224	1513	1.59	0.66
*δ*‐Cadinene	1519.18367	1519.18367	1518	0.35	2.03
*α*‐Calamenene	1524.44898	1524.16327	1523	0.08	0.02
*β*‐Calamenene	1531.91837	1532.00000	1528	0.06	0.01
*β*‐Calacorene	0.00	1533.79592	1532	0.00	0.03
*α*‐Calacorene	1538.20408	1538.20408	1536	0.05	0.11
Elemol	1549.26531	1549.42857	1547	0.57	5.31
Germacrene B	1554.89796	0.00	1558	0.08	0.00
Spathulenol	1578.48980	1578.53061	1575	0.17	1.70
Humulene epoxide II	0.00	1613.78723	1612	0.00	0.15
Cubebol	0.00	1624.93617	1623	0.00	0.04
1‐epi‐Cubenol	0.00	1629.82979	1628	0.00	0.01
*γ*‐Eudesmol	0.00	1634.04255	1632	0.00	0.40
*τ*‐Muurolol	0.00	1644.80851	1642	0.00	0.42
Cubenol	0.00	1645.70213	1646	0.00	0.26
Caryophyllene oxide	1584.57143	0.00	1580	0.72	0.00
*τ*‐Muurolol	1645.91489	0.00	1642	0.05	0.00
*α*‐Cadinol	1657.65957	1657.57447	1654	0.18	1.92
epi‐*α*‐Cadinol	0.00	1676.04255	1674	0.00	0.04
Artifact/contaminant	0.00	838.33879	—	0.00	0.06
Artifact/contaminant	0.00	931.61150	—	0.00	0.03
Artifact/contaminant	0.00	971.77155	—	0.00	0.03
Total				100	100

*Note*: RI_exp_, experimental retention index calculated relative to *n*‐alkanes (C7–C40) on an HP‐5MS column; RI_lit_, retention index reported in the literature (Adams, 2007)^17^; Area (%), relative peak area expressed as percentage of the total chromatographic area.

CC, Ceylon citronella; CJ, Java citronella.

### 3.2 Osmotic potential of essential oils and germination analysis using PEG‐6000

**Figure 1:**
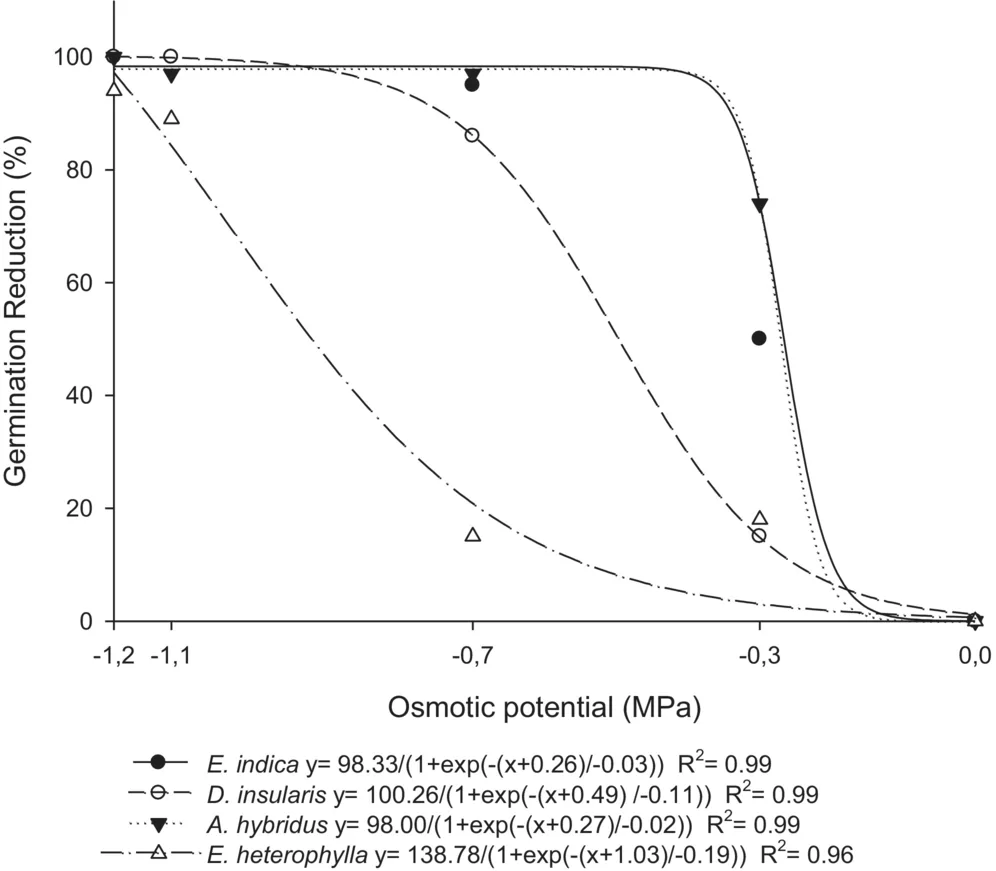
Germination of *Eleusine indica*, *Digitaria insularis*, *Amaranthus hybridus*, and *Euphorbia heterophylla* exposed to polyethylene glycol solutions corresponding to the osmotic potentials generated by the different essential oils.

The osmotic pressure values of the EOs (Table 3) indicate that, at higher concentrations, the osmotic potentials were sufficiently negative to interfere with the germination process. The species exhibited distinct levels of sensitivity to osmotic stress. In PEG‐6000 solutions at −0.30 MPa, germination reduction reached 77.8% for *Eleusine indica*, 80.1% for *A. hybridus*, 18% for *D. insularis* and only 3% for *Euphorbia heterophylla*. At −0.7 MPa, germination was reduced by 21% in *Euphorbia heterophylla*, 87% in *D*. *insularis*, and completely inhibited (98%) in *A*. *hybridus* and *Eleusine indica*. Osmotic potentials of −1.1 and − 1.2 MPa drastically reduced germination in all species. These results confirm the greater tolerance of *Euphorbia heterophylla* to osmotic stress, supporting the allelopathic potential of the EOs against this species, whereas *A*. *hybridus* and *Eleusine indica* were the most sensitive (Fig. 1).

**Table 3:** Osmotic potential values of the essential oils (EOs): orange essential oil obtained by steam distillation (LV), orange essential oil obtained by hydrodistillation (LH), Ceylon citronella (CC), and Java citronella (CJ), expressed in MPa

Concentration (mL L^−1^)	Orange EO – steam distillation (MPa) (LV)	Orange EO – hydrodistillation (MPa) (LH)	Ceylon citronella EO (MPa) (CC)	Java citronella EO (MPa) (CJ)
10	−0.28	−0.30	−0.27	−0.29
25	−0.70	−0.75	−0.69	−0.74
40	−1.12	−1.12	−1.12	−1.12
60	−1.68	−1.18	−1.16	−1.17

### 3.3 Effects of essential oils on weed germination

**Figure 2:**
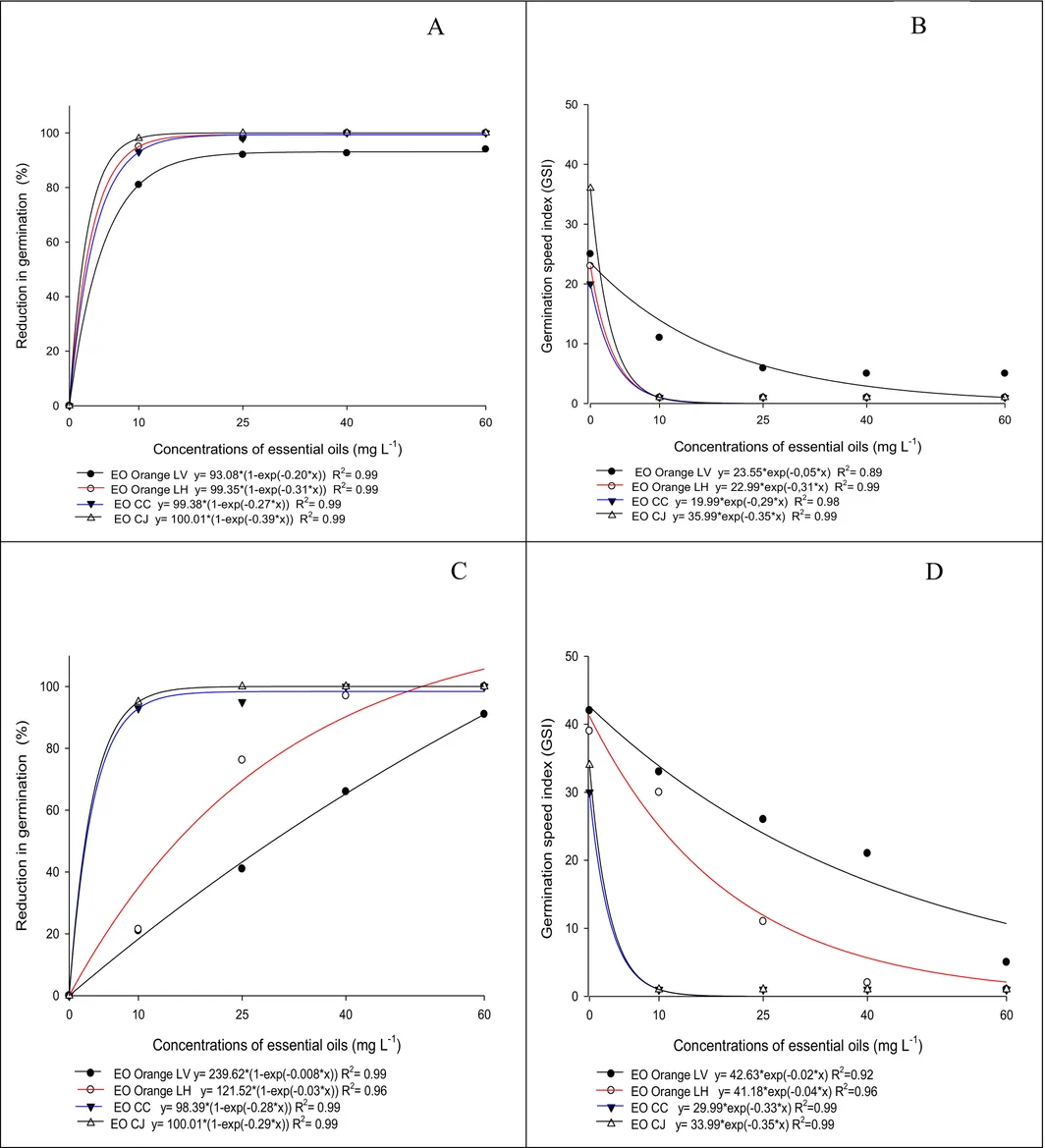
Reduction in germination and germination speed index (GSI) of *Eleusine indica* (A, B) and *Digitaria insularis* (C, D) exposed to different concentrations (0, 10, 25, 40, and 60 mL L^−1^) of essential oils obtained from orange by steam distillation, orange by hydrodistillation, Ceylon citronella and Java citronella.

Germination of *Eleusine indica* decreased with increasing EO concentrations (Fig. 2(A)). From 10 mL L^−1^ onward, Ceylon (CC) and Java (CJ) citronella EOs caused near‐complete inhibition (>90%), whereas for orange EOs, at 10 mL L^−1^ the orange steam‐distilled EO (LV) reduced germination by 80% and the orange hydrodistilled EO (LH) by 94.9%. At 25 mL L^−1^, the LV treatment resulted in a 92% reduction in germination. GSI followed the same pattern, with CC and CJ causing an early and sharp decline to values close to 0, whereas LH and LV promoted more gradual reductions, with LV maintaining higher GSI values up to 25 mL L^−1^ (Fig. 2(B)).

For *D*. *insularis* (Fig. 2(C)), citronella EOs (CC and CJ) promoted almost complete germination inhibition (92%) already at 10 mL L^−1^ and sharply reduced GSI at the lowest concentration, indicating rapid suppression of germination. By contrast, orange EOs showed weaker and more gradual effects, with LH consistently more effective than LV; regarding GSI, the greatest reductions were observed for citronella oils, whereas orange oils caused a gradual decrease as concentrations increased, with LH promoting greater reduction than LV (Fig. 2(D)).

**Figure 3:**
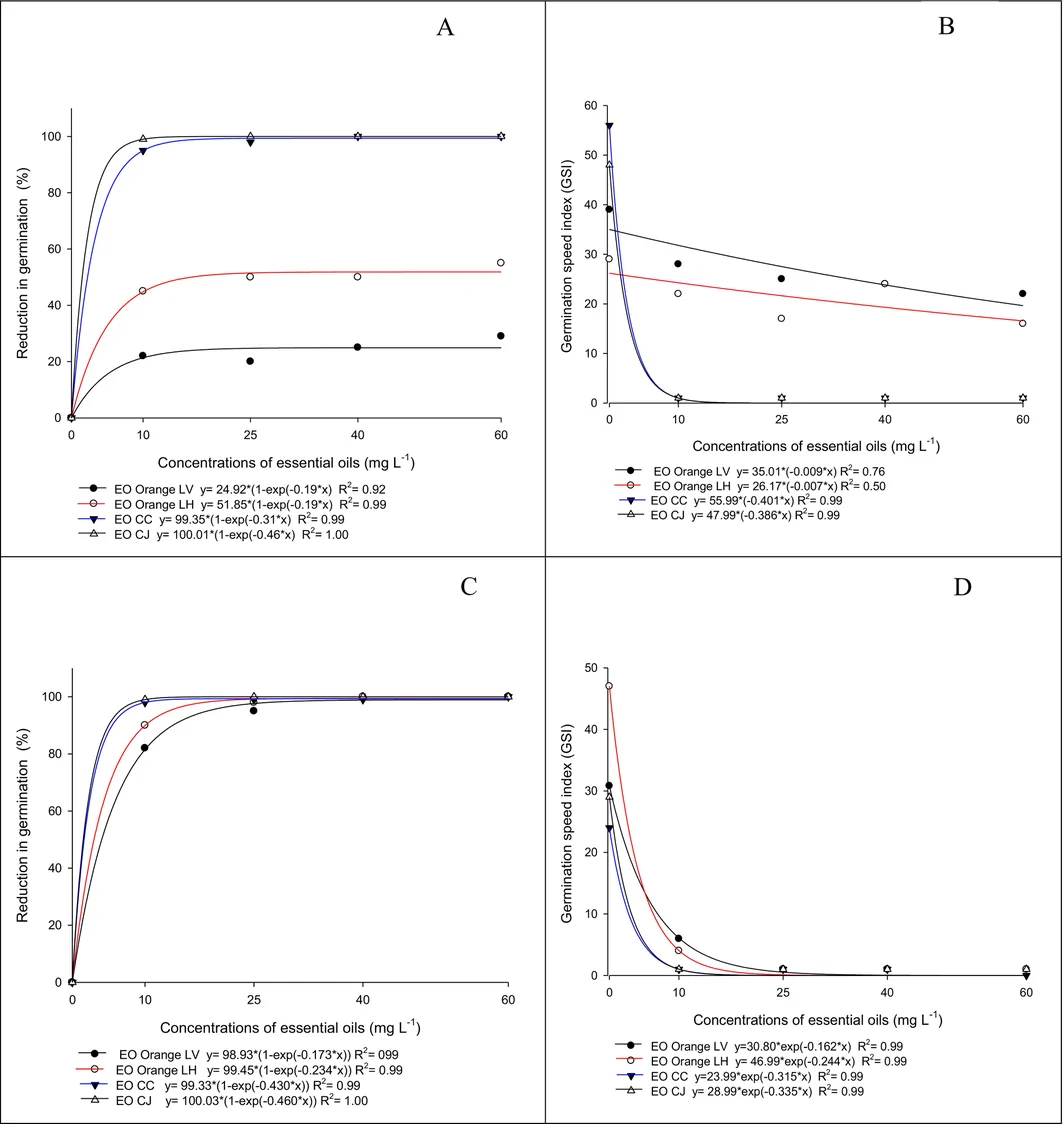
Reduction in germination and germination speed index (GSI) of *Euphorbia heterophylla* (A, B) and *Amaranthus hybridus* (C, D) exposed to different concentrations (0, 10, 25, 40, and 60 mL L^−1^) of essential oils obtained from orange by steam distillation, orange by hydrodistillation, Ceylon citronella and Java citronella.

Germination of *Euphorbia heterophylla* declined with increasing EO concentrations (Fig. 3(A)). Citronella EOs (CC and CJ) caused near‐total inhibition from 10 mL L^−1^ onward, whereas the orange EO obtained by hydrodistillation (LH) reduced germination by approximately 44% at the highest concentration and the steam‐distilled orange EO (LV) showed limited inhibition (21%). GSI responses (Fig. 3(B)) mirrored germination patterns, with CC and CJ reaching values close to zero, while LH and LV maintained higher GSI values and lower model fit (*R* ^2^ = 0.50 and 0.76).

For *A*. *hybridus*, germination was reduced by 81.4% and 89.9% at 10 mL L^−1^ for LV and LH, respectively, and by more than 99% for CC and CJ (Fig. 3(C)). From 25 mL L^−1^ onward, germination reduction was virtually complete for all treatments. Accordingly, GSI declined sharply for CC and CJ from 10 mL L^−1^ onward, while LH showed intermediate effects and LV the most gradual decline (Fig. 3(D)).

### 3.4 Effect of essential oils on weed growth: BOD chamber

For *Eleusine indica*, no statistically significant differences were observed in SL or RL reduction among treatments (Table 4). In *D*. *insularis*, the orange LV caused the greatest SL reduction (23.90%) at 10 mL L^−1^, differing significantly from the other treatments, indicating sensitivity to citrus compounds, likely associated with limonene. Citronella EOs (CC and CJ) showed moderate effects on SL. For RL, CJ was the most effective treatment at 10 mL L^−1^ (35.02%), followed by LV, while higher concentrations resulted in weaker responses (Table 4).

**Table 4:** Percentage reduction of shoot length and root length of *Eleusine indica* and *Digitaria insularis* in the weed early growth experiment

Concentration (mL L^−1^)	LV	LH	CC	CJ
Percentage reduction of shoot length of *E*. *indica*				
0	0.00 Aa	0.00 Aa	0.00 Aa	0.00 Aa
10	12.64 Aa	8.42 Aa	6.05 Aa	7.22 Aa
25	9.03 Aa	15.68 Aa	0.27 Aa	10.00 Aa
40	0.00 Aa	6.02 Aa	1.35 Aa	13.01 Aa
60	2.97 Aa	6.44 Aa	3.74 Aa	11.72 Aa
Percentage reduction of root length of *E*. *indica*				
0	0.00 Aa	0.00 Aa	0.00 Aa	0.00 Aa
10	15.67 Aa	9.02 Aa	3.22 Aa	13.64 Aa
25	10.98 Aa	18.06 Aa	0.00 Aa	2.08 Aa
40	12.13 Aa	12.61 Aa	6.16 Aa	3.89 Aa
60	11.07 Aa	15.00 Aa	3.44 Aa	5.76 Aa
Percentage reduction of shoot length of *D*. *insularis*				
0	0.00 Ab	0.00 Ab	0.00 Ab	0.00 Ab
10	23.90 Aa	10.36 Ba	9.19 Ba	13.98 Ba
25	0.40 Bb	12.05 Aa	0.00 Bb	0.00 Bb
40	1.09 Ab	4.93 Aab	3.72 Aab	5.58 Aa
60	4.02 Ab	6.63 Aab	4.26 Aab	0.00 Ab
Percentage reduction of root length of *D*. *insularis*				
0	0.00 Ab	0.00 Aa	0.00 Aa	0.00 Ab
10	29.94 Aa	12.35 Ba	14.65 Ba	35.02 Aa
25	1.19 Ab	9.26 Aa	1.10 Aa	0.00 Ab
40	0.25 Ab	8.00 Aa	4.67 Aa	11.18 Ab
60	5.05 Ab	12.31 Aa	8.16 Aa	5.13 Ab

*Note*: Means followed by the same lowercase letter within a column and the same uppercase letter within a row do not differ significantly according to Tukey's test at the 5% significance level. CV SL (%) = 127.1% (*E*. *indica*) and 91.7% (*D*. *insularis*). CV RL (%) = 163.4% (*E*. *indica*) and 93.2% (*D*. *insularis*).

CC, Ceylon citronella oil; CJ, Java citronella oil; LH, hydrodistilled orange oil; LV, steam‐distilled orange oil.

In *Euphorbia heterophylla*, the strongest SL reductions occurred at 10 mL L^−1^ for LV (49.95%), CJ (49.81%) and CC (46.50%), indicating pronounced early phytotoxicity; for LH, there was no significant difference compared with the control. At higher concentrations, SL reductions were less pronounced. Root growth was particularly sensitive, with CC (58.06%), CJ (57.27%) and LV (51.74%) causing substantial RL reductions at 10 mL L^−1^ (Table 5).

**Table 5:** Percentage reduction of shoot length and root length of *Euphorbia heterophylla* and *Amaranthus hybridus* in the weed early growth experiment

Concentration (mL L^−**1**^)	LV	LH	CC	CJ
Percentage reduction of shoot length of *E*. *heterophylla*				
0	0.00 Ab	0.00 Aa	0.00 Ab	0.00 Ab
10	49.95 Aa	13.63 Ba	46.50 Aa	49.81 Aa
25	25.28 Aa	23.18 Aa	2.87 Ab	6.85 Ab
40	0.00 Bb	29.80 Aa	4.53 ABb	16.27 ABb
60	7.00 Ab	9.01 Aa	0.00 Ab	1.23 Ab
Percentage reduction of root length of *E*. *heterophylla*				
0	0.00 Ab	0.00 Ab	0.00 Ab	0.00 Ab
10	51.74 Aa	28.74 Ba	58.06 Aa	57.27 Aa
25	21.41 ABb	29.58 Aa	1.60 Bb	2.36 Bb
40	0.00 Bb	30.51 Aa	1.17 Bb	15.59 ABb
60	5.71 Ab	3.49 Ab	1.13 Ab	0.00 Ab
Percentage reduction of shoot length of *A*. *hybridus*				
0	0.00 Aa	0.00 Ab	0.00 Aa	0.00 Aa
10	6.04 Ba	18.90 Aa	1.56 Ba	10.15 Aba
25	2.54 Ba	14.97 Aa	5.70 Aba	1.93 Ba
40	3.68 Aa	3.06 Ab	5.45 Aa	4.17 Aa
60	1.94 Aa	2.91 Ab	0.57 Aa	3.62 Aa
Percentage reduction of root length of *Amaranthus hybridus*				
0	0.00 Aa	0.00 Aa	0.00 Aa	0.00 Aa
10	13.82 Aa	1.81 Aa	4.47 Aa	13.82 Aa
25	15.75 Aa	4.44 Aa	3.32 Aa	15.75 Aa
40	0.76 Aa	8.73 Aa	4.88 Aa	0.76 Aa
60	0.67 Aa	4.63 Aa	3.26 Aa	0.67 Aa

*Note*: Means followed by the same lowercase letter within a column and the same uppercase letter within a row do not differ significantly according to Tukey's test at the 5% significance level. CV RL (%) = 74.9% (*E*. *heterophylla) and* 150.9% (*A*. *hybridus*).

CC, Ceylon citronella oil; CJ, Java citronella oil; LH, hydrodistilled orange oil; LV, steam‐distilled orange oil.

For *A*. *hybridus*, the orange EO (LH) caused the greatest reductions, decreasing SL by 18.90% at 10 mL L^−1^ and 14.97% at 25 mL L^−1^ and RL by 15.75% at 25 mL L^−1^, indicating sensitivity to volatile citrus compounds (Table 5).

### 3.5 Post‐emergence phytotoxicity of essential oils

**Figure 4:**
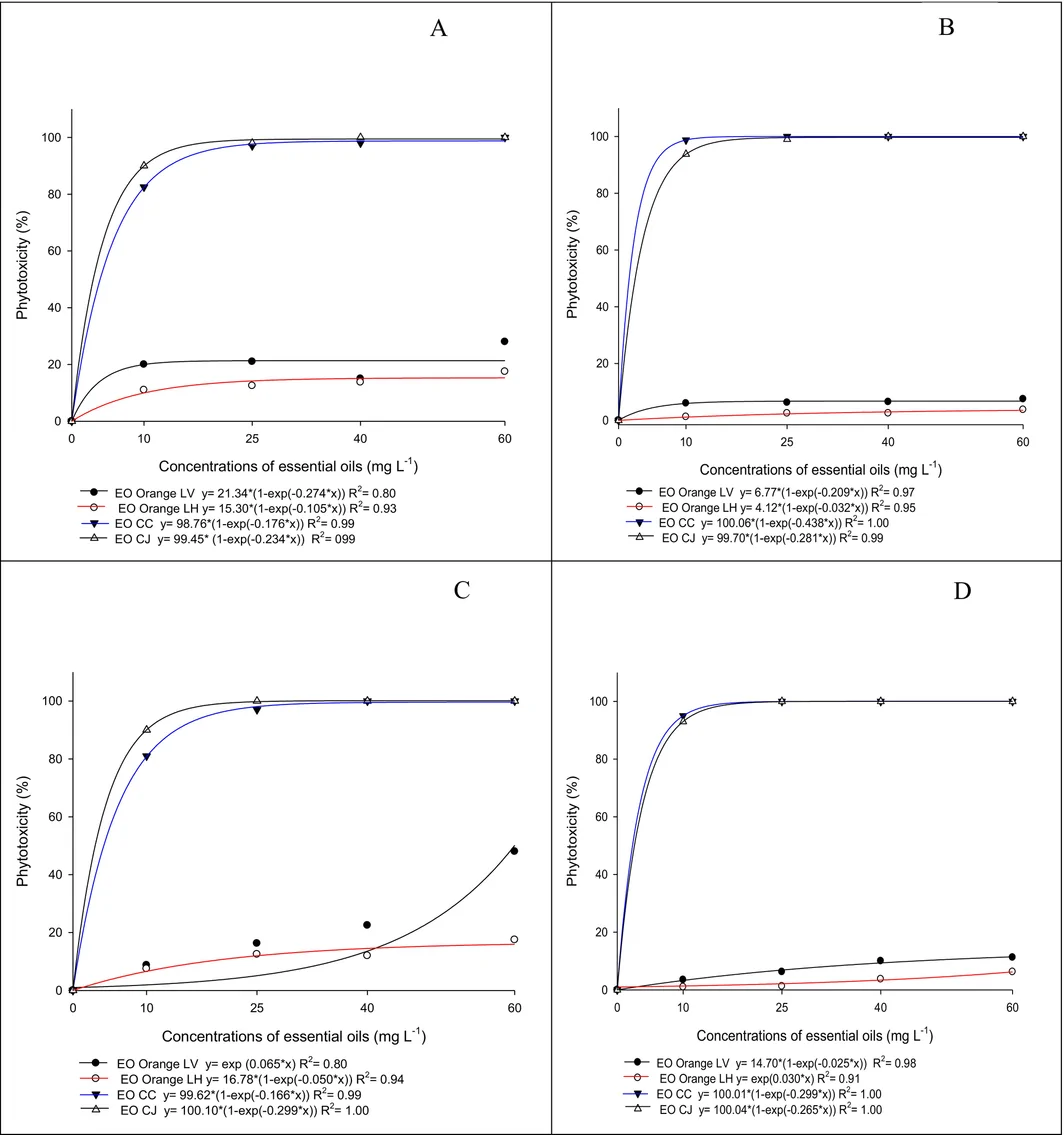
Phytotoxicity at 8 and 20 days after application (DAA), respectively, of *Eleusine indica* (A, B) and *Digitaria insularis* (C, D) exposed to different concentrations (0, 10, 25, 40, and 60 mL L^−1^) of essential oils obtained from orange by steam distillation (LV), orange by hydrodistillation (LH), Ceylon citronella (CC) and Java citronella (CJ).

**Figure 5:**
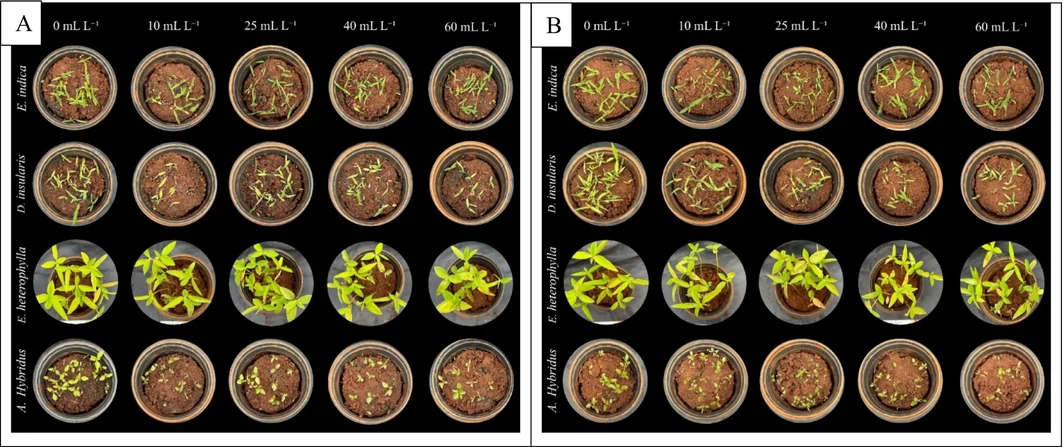
Phytotoxic effect of orange essential oil obtained by hydrodistillation (A) and orange essential oil obtained by steam distillation (B) on seedlings of *Eleusine indica*, *Digitaria insularis*, *Euphorbia heterophylla* and *Amaranthus hybridus* at different concentrations (0, 10, 25, 40, and 60 mL L^−1^), evaluated 20 days after application.

**Figure 6:**
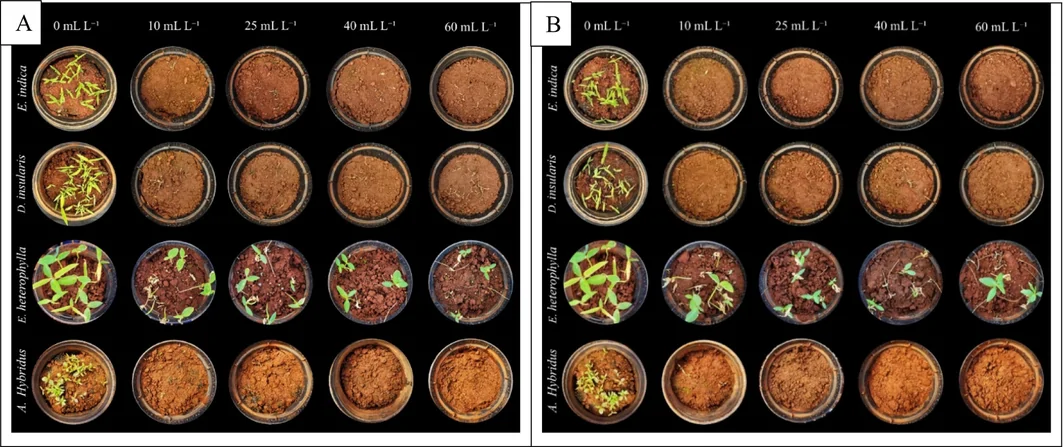
Phytotoxic effect of citronella essential oil obtained by Ceylon citronella (A) and Java citronella (B) on seedlings of *Eleusine indica*, *Digitaria insularis*, *Euphorbia heterophylla* and *Amaranthus hybridus* at different concentrations (0, 10, 25, 40 and 60 mL L^−1^), evaluated 20 days after application.

Post‐emergence evaluations revealed clear differences among EOs and weed species. In *Eleusine indica*, CC and CJ citronella EOs caused high phytotoxicity (>80%) even at the lowest concentrations and maintained complete control at 20 DAA, with no evidence of seedling recovery (Figs 4, 5, 6). By contrast, orange EOs (LH and LV) showed low post‐emergence efficacy, with phytotoxicity below 20% and progressive recovery by 20 DAA.

A similar response pattern was observed for *D*. *insularis* (Figs 4, 5, 6). CC and CJ oils induced rapid and sustained phytotoxicity, exceeding 80% at 8 DAA and maintaining near‐total control at 20 DAA. Conversely, LH and LV exhibited weak and transient effects (<6.6% at 10 mL L^−1^), with declining phytotoxicity over time, indicating insufficient post‐emergence control.

**Figure 7:**
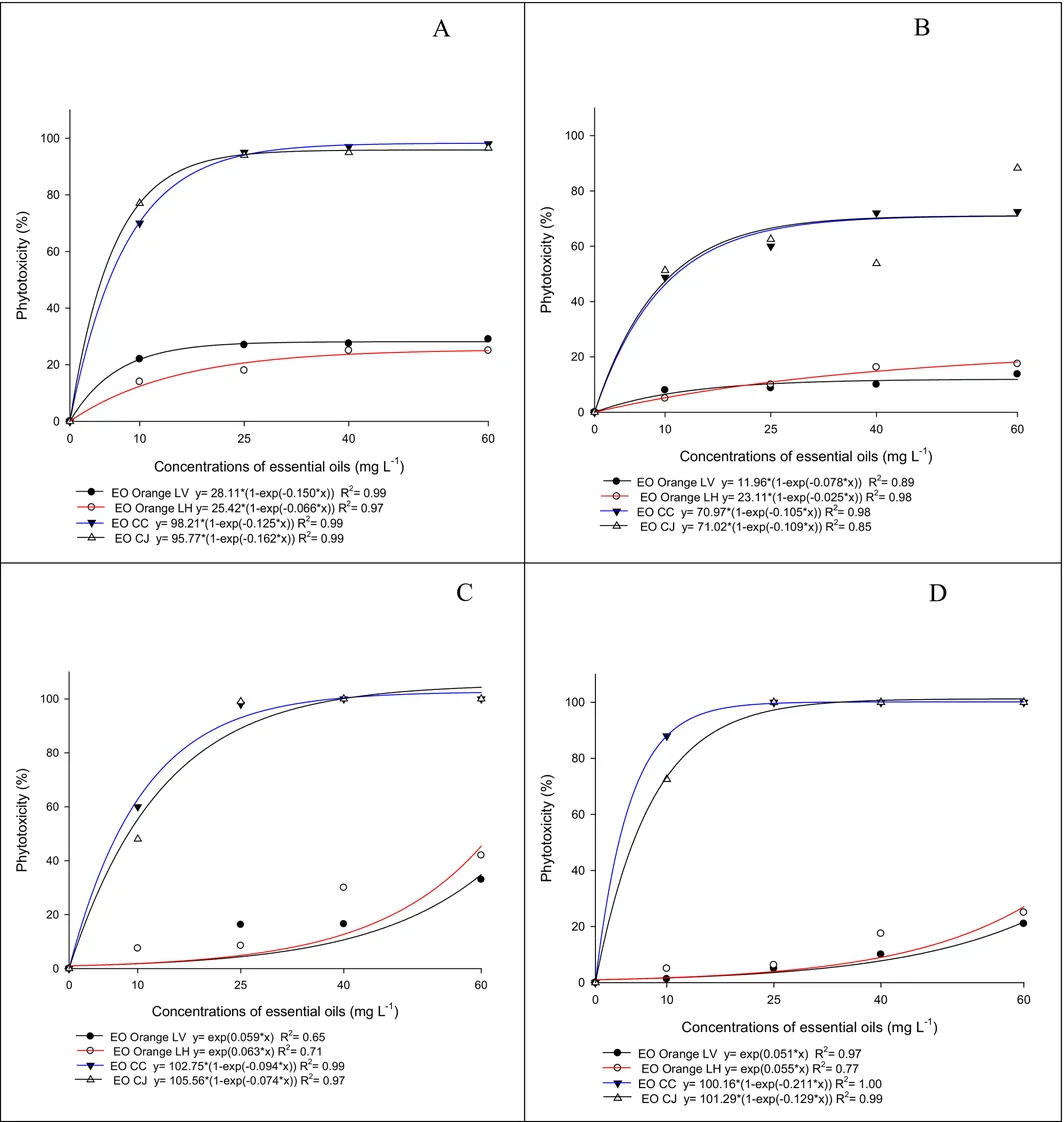
Phytotoxicity at 8 and 20 days after application, respectively, of *Euphorbia heterophylla* (A, B) and *Amaranthus hybridus* (C, D) exposed to different concentrations (0, 10, 25, 40, and 60 mL L^−1^) of essential oils obtained from orange by steam distillation (LV), orange by hydrodistillation (LH), Ceylon citronella (CC) and Java citronella (CJ).

For *Euphorbia heterophylla*, at 8 DAA, CC and CJ treatments resulted in control levels ranging from 70.0% to 93.8% and from 76.8% to 94.1%, respectively, at doses of 10 to 25 mL L^−1^, indicating a clear dose‐dependent effect. However, at the 20 DAA evaluation, control reached a maximum of only 70% at the highest dose (Fig. 7). For orange EOs, a similar trend of reduced phytotoxicity was observed between the 8 and 20 DAA evaluations, with maximum control of 24% and 17% for LH and 28% and 11% for LV at 8 and 20 DAA, respectively.

In *A*. *hybridus*, CC and CJ demonstrated rapid and persistent phytotoxic effects, reaching near‐complete control at higher concentrations with no recovery at 20 DAA (Fig. 7). By contrast, LH and LV caused only moderate injury, with maximum phytotoxicity between 25% and 35%, followed by seedling recovery.

The images in Figs 5 and 6 allow the observation of symptoms such as leaf yellowing, necrosis and reduced weed development in response to different EO concentrations, with these effects being more pronounced in the citronella treatments.

Evaluation of SL and RL reduction showed that CC and CJ citronella EOs were consistently the most effective treatments, whereas orange EOs obtained by LV and LH exhibited moderate and concentration‐dependent effects.

For *Eleusine indica* and *D*. *insularis*, CC and CJ caused complete inhibition (100%), indicating strong phytotoxic activity. For *Eleusine indica*, LV and LH showed low effects on SL and RL. The best result was observed at the 60 mL L^−1^ dose for the LH treatment, with a reduction of 54.75%, statistically superior to the other doses, but inferior to CC and CJ. For *D*. *insularis*, greater suppression was observed in RL compared with SL, with effects becoming more pronounced at higher doses. The most effective treatment for this species was LH at the 40 mL L^−1^ dose on RL, resulting in a 70.17% reduction (Table 6).

**Table 6:** Percentage reduction of shoot length and root length of *Eleusine indica* and *Digitaria insularis* in the post‐emergence phytotoxicity experiment.

Concentration (mL L^−**1**^)	LV	LH	CC	CJ
Percentage reduction of shoot length of *E*. *indica*				
0	0.00 Ab	0.00 Ac	0.00 Ab	0.00 Ab
10	1.55 Bb	6.88 Bbc	100 Aa	100 Aa
25	20.29 Bab	15.26 Bbc	100 Aa	100 Aa
40	19.49 Bab	26.24 Bb	100 Aa	100 Aa
60	33.40 Ca	54.75 Ba	100 Aa	100 Aa
Percentage reduction of root length of *E*. *indica*				
0	0.00 Ab	0.00 Ac	0.00 Ab	0.00 Ab
10	19.15 Ba	22.08 Bb	100 Aa	100 Aa
25	19.18 Ca	30.81 Bab	100 Aa	100 Aa
40	19.08 Ca	34.67 Ba	100 Aa	100 Aa
60	22.77 Ba	31.25 Ba	100 Aa	100 Aa
Percentage reduction of shoot length of *D*. *insularis*				
0	0.00 Ab	0.00 Ac	0.00 Ab	0.00 Ab
10	13.54 Bb	7.29 Bbc	100 Aa	100 Aa
25	31.36 Ba	26.37 Bab	100 Aa	100 Aa
40	39.93 Ba	21.33 Cab	100 Aa	100 Aa
60	38.69 Ba	29.71 Ba	100 Aa	100 Aa
Percentage reduction of root length of *D*. *insularis*				
0	0.00 Ad	0.00 Ad	0.00 Ab	0.00 Ab
10	30.18 Bc	31.25 Bc	100 Aa	100 Aa
25	61.54 Bb	62.25 Bb	100 Aa	100 Aa
40	69.03 Ba	70.17 Ba	100 Aa	100 Aa
60	66.88 Ba	67.78 Ba	100 Aa	100 Aa

*Note*: Means followed by the same lowercase letter within a column and the same uppercase letter within a row do not differ significantly according to Tukey's test at the 5% significance level. CV SL (%) = 18.3% (*E*. *indica*) and 15.9% (*D*. *insularis*). CV RL (%) = 9.16% (*E*. *indica*) and 4.22% (*D*. *insularis*).

CC, Ceylon citronella oil; CJ, Java citronella oil; LH, hydrodistilled orange oil; LV, steam‐distilled orange oil.

For *Euphorbia heterophylla*, CC and CJ also promoted high levels of inhibition, with reductions of up to 73.53% in SL and 88.55% in RL, indicating severe impairment of seedling growth (Table 7). CC and CJ caused complete inhibition (100%) for *A. hybridus*. In *Euphorbia heterophylla* LV and LH oils were less effective, although LV did not result in a significant difference between the doses for RL, with maximum reductions of 25.57% (SL) and 35.23% (RL), although visual symptoms such as seedling deformities were observed. *A*. *hybridus* showed a reduction of 68.55% (LV) and 70.81% (LH) for RL at the highest dose (60 mL L^−1^).

**Table 7:** Percentage reduction of shoot length and root length of *Euphorbia heterophylla* and *Amaranthus hybridus* in the post‐emergence phytotoxicity experiment

Concentration (mL L^−**1**^)	LV	LH	CC	CJ
Percentage reduction of shoot length of *E*. *heterophylla*				
0	0.00 Ab	0.00 Ab	0.00 Ac	0.00 Ac
10	4.47 Bb	0.00 Bb	27.48 Ab	34.15 Ab
25	21.98 Ba	7.06 Cb	67.07 Aa	69.47 Aa
40	20.18 Ba	6.76 Cb	71.71 Aa	73.53 Aa
60	25.57 Ba	18.88 Ba	70.66 Aa	71.67 Aa
Percentage reduction of root length of *E*. *heterophylla*				
0	0.00 Aa	0.00 Ab	0.00 Ab	0.00 Ac
10	3.66 Ba	15.00 Bab	67.48 Aa	71.48 Aab
25	9.23 Ba	21.06 Bab	75.98 Aa	79.15 Aab
40	8.28 Ca	27.50 BCab	82.45 Aa	46.52 Bb
60	27.41 Ba	35.23 Ba	88.06 Aa	88.55 Aa
Percentage reduction of shoot length of *A*. *hybridus*				
0	0.00 Ad	0.00 Ad	0.00 Ab	0.00 Ab
10	36.63 Bc	26.29 Cc	100 Aa	100 Aa
25	44.84 Bb	36.72 Cb	100 Aa	100 Aa
40	45.59 Bb	41.61 Bb	100 Aa	100 Aa
60	62.95 Ba	59.87 Ba	100 Aa	100 Aa
Percentage reduction of root length of *A*. *hybridus*				
0	0.00 Ae	0.00 Ae	0.00 Ab	0.00 Ab
10	8.71 Bd	7.95 Bd	100 Aa	100 Aa
25	14.05 Bc	14.34 Bc	100 Aa	100 Aa
40	53.33 Bb	53.80 Bb	100 Aa	100 Aa
60	68.55 Ba	70.81 Ba	100 Aa	100 Aa

*Note*: Means followed by the same lowercase letter within a column and the same uppercase letter within a row do not differ significantly according to Tukey's test at the 5% significance level. CV RL (%) = 44.37% (*E*. *heterophylla*) and 4.5% (*A*. *hybridus*).

CC, Ceylon citronella oil; CJ, Java citronella oil; LH, hydrodistilled orange oil; LV, steam‐distilled orange oil.

Regarding biomass accumulation, CC and CJ caused complete suppression in *Eleusine indica* from 10 mL L^−1^ onward, resulting in the absence of dry matter accumulation (Table 8). LV and LH induced progressive biomass reductions, with the lowest values at the highest concentrations. A similar pattern was observed for *D*. *insularis*, with null biomass for CC and CJ from 10 mL L^−1^ onward and gradual reductions for LV and LH.

**Table 8:** Biomass of *Eleusine indica* and *Digitaria insularis* subjected to different concentrations of essential oils (0, 10, 25, 40, and 60 mL L^−1^)

Concentration (mL L^−1^)	LV	LH	CC	CJ
Biomass reduction of *E*. *indica*				
0	0.25 Aa	0.13 Aa	0.42 Aa	0.34 Aa
10	0.18 Aab	0.11 ABa	0.00 Bb	0.00 Bb
25	0.06 Ab	0.10 Aa	0.00 Ab	0.00 Ab
40	0.07 Aab	0.06 Aa	0.00 Ab	0.00 Ab
60	0.09 Aab	0.06 Aa	0.00 Ab	0.00 Ab
Biomass reduction of *D*. *insularis*				
0	0.21 Aa	0.22 Aa	0.22 Aa	0.21 Aa
10	0.08 Ab	0.08 Ab	0.00 Bb	0.00 Bb
25	0.05 Ab	0.04 ABb	0.00 Bb	0.00 Bb
40	0.04 Ab	0.04 Ab	0.00 Ab	0.00 Ab
60	0.04 ABb	0.05 Ab	0.00 Bb	0.00 Bb

*Note*: Means followed by the same lowercase letter within a column and the same uppercase letter within a row do not differ significantly according to Tukey's test at the 5% significance level. CV (%) = 100.2% (*E*. *indica*) and 45.46% (*D*. *insularis*).

CC, Ceylon citronella oil; CJ, Java citronella oil; LH, hydrodistilled orange oil; LV, steam‐distilled orange oil.

In *Euphorbia heterophylla*, CC and CJ drastically reduced biomass, reaching values close to zero at the highest concentrations, whereas LV and LH maintained higher biomass values, indicating lower phytotoxic intensity and statistically different from CC and CJ (Table 9). For *A*. *hybridus*, CC and CJ also caused complete biomass suppression from 10 mL L^−1^ onward, whereas LV and LH promoted substantial, though less intense, growth inhibition.

**Table 9:** Biomass of *Euphorbia heterophylla* and *Amaranthus hybridus* subjected to different concentrations of essential oils (0, 10, 25, 40, and 60 mL L^−1^)

Concentration (mL L^−**1**^)	LV	LH	CC	CJ
Biomass reduction of *E*. *heterophylla*				
0	0.63 Aa	0.62 Aa	0.30 Aa	0.31 Aa
10	0.51 Ab	0.57 Ab	0.09 Bb	0.07 Bb
25	0.48 Ab	0.49 Ab	0.06 Bb	0.04 Bb
40	0.46 Ab	0.47 Ab	0.05 Bb	0.01 Bb
60	0.44 Ab	0.46 Ab	0.02 Bb	0.00 Bb
Biomass reduction of *A*. *hybridus*				
0	0.10 Aa	0.09 Aa	0.10 Aa	0.11 Aa
10	0.05 Ab	0.05 Ab	0.00 Bb	0.00 Bb
25	0.06 Ab	0.05 Ab	0.00 Bb	0.00 Bb
40	0.03 Ab	0.04 Ab	0.00 Ab	0.00 Ab
60	0.02 Ab	0.02 Ab	0.00 Ab	0.00 Ab

*Note*: Means followed by the same lowercase letter within a column and the same uppercase letter within a row do not differ significantly according to Tukey's test at the 5% significance level. CV (%) = 32.7% (*E*. *heterophylla*) and 54.6% (*A*. *hybridus*).

CC, Ceylon citronella oil; CJ, Java citronella oil; LH, hydrodistilled orange oil; LV, steam‐distilled orange oil.

## 4 DISCUSSION

### 4.1 Chromatographic analysis of essential oils

The phytotoxic responses observed in this study are closely linked to the chemical composition of the EOs. Citronella EOs were characterized by high proportions of oxygenated monoterpenes, particularly citronellol, citronellal, geraniol and citral isomers, compounds widely associated with herbicidal activity. According to Kaur *et al*.,^27^ citronellol has been reported to induce strong inhibition of root and shoot growth through mechanisms involving reactive oxygen species generation, lipid peroxidation, membrane damage, solute leakage and cell death.

Supporting these findings, Lins *et al*.^28^ demonstrated that citronellol and citronellal interact selectively with biomimetic membranes, altering lipid organization and membrane domains, even in the absence of immediate permeabilization. Such physicochemical changes may disrupt membrane‐dependent metabolic processes, reinforcing the role of these compounds as natural contact herbicides. This mode of action is fundamentally different from systemic herbicides like glyphosate, which act through specific enzymatic pathways. Instead, the activity of citronella‐derived monoterpenes is more consistent with contact herbicides, such as pelargonic acid, where the plasma membrane emerges as a primary cellular target. Thus, the disruption of lipid bilayer stability appears to be the central mechanism of the phytotoxicity observed for these monoterpenes.

In addition to membrane‐related effects, Chaimovitsh *et al*.^29^ reported that monoterpenes may disrupt the plant cytoskeleton, particularly microtubules involved in cell division and elongation. Limonene and citronellal exhibited strong antimicrotubular activity, whereas geraniol and citronellol showed more moderate effects. The biotransformation of limonene into carvacrol in plants suggests a dual mode of action involving both microtubule disorganization and membrane disruption, which may contribute to its biological activity. Although these mechanisms were not directly assessed in this study, they are consistent with the observed phytotoxic responses and with previous literature.

Consistent with these mechanisms, Azirak and Karaman^30^ reported strong inhibition of weed germination by carvacrol, thymol, carvone and limonene, even at low concentrations. Collectively, these studies support the conclusion that the phytotoxic effects of EOs are primarily driven by their monoterpene composition, which acts on multiple cellular targets, including membranes, microtubules and oxidative balance.

For orange EO, the LV extraction method resulted in the identification of 18 compounds not detected in LH. However, despite these compositional differences, both oils exhibited comparatively lower phytotoxicity, suggesting that the extraction method had a limited effect on weed control efficacy.

### 4.2 Osmotic potential of essential oils and use of PEG‐6000

Osmotic potential is a critical factor regulating seed germination, because it directly affects water uptake and metabolic reactivation of the embryo. Therefore, the use of PEG‐6000 as an osmotic control was essential to distinguish between true allelochemical effects of EOs and possible osmotic stress‐induced inhibition.^31^

Previous studies indicate that germination and early seedling development are significantly affected only when osmotic potentials fall outside the range of −0.2 to −0.6 MPa.^32^, ^33^ Thus, the PEG‐based approach adopted in this study provides a framework to compare osmotic and non‐osmotic effects, although additional physiological and biochemical analyses would be necessary to fully disentangle these mechanisms.

Considering that osmotic potentials below −0.7 MPa are sufficient to strongly inhibit seed germination, part of the inhibitory effects observed in the EO treatments may be associated with osmotic stress. However, citronella EOs (CC and CJ) reduced weed germination even at the lowest concentration tested (10 mL L^−1^), which presented osmotic potentials around −0.3 MPa. Because osmotic potentials in this range are generally not considered severely restrictive to germination,^32^, ^33^ and the inhibition was greater than that observed in PEG‐6000 solutions with equivalent osmotic potentials, these findings indicate that biochemical interactions associated with allelochemicals also contribute to the inhibitory effects.

Therefore, the results suggest that both osmotic and biochemical factors are involved in the inhibition process, although further studies are still required to quantify the relative contribution of each mechanism.

### 4.3 Effects of essential oils on weed germination

CC and CJ were consistently the most effective treatments, promoting near‐complete inhibition of germination and GSI at low concentrations. This high bioactivity is attributed to their enrichment in oxygenated monoterpenes, such as citronellal, geraniol and citronellol. For the LH and LV treatments, responses varied among species, with reduced effectiveness against *Euphorbia heterophylla* and *D*. *insularis*, suggesting a lower potential for pre‐emergence application on these weeds. Similar dose‐dependent inhibitory effects and species‐specific responses to volatile terpenoids have been reported by Abd‐ElGawad *et al*.^10^

These findings are consistent with Ribeiro and Lima,^34^ who associated the bioactivity of citrus EOs with limonene, capable of inhibiting germination of *Euphorbia heterophylla* and *Ipomoea grandifolia*. Similar inhibitory patterns were reported in different weed species exposed to citrus volatiles.^7^, ^35^

The strong inhibitory effects observed for citronella oils corroborate reports by Marco *et al*.,^36^ Corrêa *et al*.,^37^ and Borges *et al*.,^38^ who documented significant germination suppression in several plant species. The allelopathic activity of citronella has been consistently attributed to citronellal, geraniol and citronellol, which promote dose‐dependent reductions in germination and early root growth.^39^ Similar strong phytotoxic effects associated with oxygenated terpenoid‐rich EOs have been reported by Assaeed *et al*.,^12^ who observed significant inhibition of weed germination and seedling growth.

### 4.4 Effects of essential oils on weed growth

Seedlings treated with EOs exhibited visual symptoms such as albinism and root deformities, suggesting possible interference with pigment synthesis and root development. According to An *et al*.,^40^ the biological response to allelochemicals is strongly dependent on both concentration and the sensitivity of the recipient species.

The observed responses are consistent with an inverse hormetic pattern commonly reported for EOs. At low concentrations, monoterpenes may penetrate tissues more efficiently, potentially affecting membrane integrity, cell division, mitochondrial respiration and hormonal balance.^41^

Roots, as the primary point of contact, were particularly sensitive, which may explain the greater reductions in RL. Similar responses have been reported by Abd‐ElGawad *et al*.,^9^ who observed that EOs rich in terpenes strongly inhibited seedling development, with root growth being the most affected parameter.

These findings agree with Carvalhal *et al*.^15^ and Melhorança Filho *et al*.^42^, who identified root growth as a sensitive indicator of allelopathic effects. Similar morphological alterations have been reported by Dorneles *et al*.^43^ and Figueiredo,^44^ and have been associated with disruption of cell division and elongation processes. According to RAS,^20^ the abnormal root morphology observed here may reflect compromised physiological integrity.

Recent *in silico* studies also suggest that EO (*Amaranthus viridis*) constituents may interact with key enzymatic targets such as glutamine synthetase and acetohydroxyacid synthase,^11^ although such mechanisms were not evaluated in the current study.

### 4.5 Post‐emergence phytotoxicity of essential oils

Overall, citronella (CC and CJ) and orange (LV and LH) EOs exhibited phytotoxic activity by reducing shoot and root growth and biomass accumulation in the evaluated weed species. However, citronella EOs were markedly more effective, promoting sustained and, in some cases, complete growth inhibition, whereas orange EOs showed milder, concentration‐dependent effects. The results showed potential for post‐emergence use of CC and CJ against *Eleusine indica*, *D*. *insularis* and *A*. *hybridus*, with slightly lower efficacy against *Euphorbia heterophylla*. By contrast, LV and LH did not show efficacy for post‐emergence application.

These results corroborate previous studies highlighting the herbicidal potential of EOs, particularly those rich in oxygenated monoterpenes.^4^, ^7^, ^45^ The reduction in green coloration and the appearance of albino seedlings suggest possible inhibition of chlorophyll synthesis and damage to chloroplasts, as reported by Ootani *et al*.^46^ and Somala *et al*.,^47^ indicating that volatile compounds may interfere with essential physiological processes in plants.

## 5 CONCLUSIONS

The results of this study demonstrate that EOs exhibit distinct and compound‐dependent phytotoxic effects on weed germination, early growth and post‐emergence development. Citronella EOs, characterized by a high proportion of oxygenated monoterpenes, showed consistent and strong bioactivity across all evaluated species, resulting in rapid germination inhibition, severe growth suppression and sustained post‐emergence phytotoxicity. By contrast, orange EOs, dominated by limonene, displayed moderate and concentration‐dependent effects, with limited post‐emergence efficacy. This comparatively lower phytotoxicity should not be interpreted as a direct consequence of limonene predominance, because the biological response of EOs depends on multiple factors, including their physicochemical behavior and interactions among constituents. However, further studies including field validation, formulation development and persistence assessments are necessary to confirm the practical applicability of these EOs as bioherbicides.

## CONFLICT OF INTEREST

The authors declare no competing interests.

## Data Availability

The data that support the findings of this study are available from the corresponding author upon reasonable request.
